# Health Facilities' Readiness to Manage Hypertension and Diabetes Cases at Primary Health Facilities in Bidibidi Refugee Settlement, Yumbe District, Uganda

**DOI:** 10.1155/2021/1415794

**Published:** 2021-01-22

**Authors:** Vuchiri Ray Isadru, Rose Clarke Nanyonga, John Bosco Alege

**Affiliations:** ^1^Institute of Public Health and Management, Clark International University, Kampala, Uganda; ^2^School of Public Health and Applied Human Sciences, Kenyatta University, Nairobi, Kenya

## Abstract

**Background:**

NCDs are the greatest global contributors to morbidity and mortality and are a major health challenge in the 21st century. The global burden of NCDs remains unacceptably high. Access to care remains a challenge for the majority of persons living with NCDs in sub-Saharan Africa. In Uganda, 55% of refugee households, including those with chronic illnesses, lack access to health services. Of these, 56% are in the West-Nile region where the Bidibidi settlement is located, with 61% of its refugee households in need of health services especially for NCDs (UNHCR, 2019). Data on NCDs in Bidibidi are scarce. Unpublished health facilities' (HFs) data indicate that cardiovascular diseases (CVDs) (54.3%) and metabolic disorders (20.6%) were the leading causes of consultation for major NCDs (IRC, 2019). No readiness assessment has ever been conducted to inform strategies for the efficient management of NCDs to avert more morbidity, mortality, and the economic burden associated with NCD management or complications among refugees. This study sought to determine the readiness of HFs in managing hypertension (HTN) and diabetes cases at primary health facilities in the Bidibidi refugee settlement, Yumbe district, Uganda.

**Methods:**

The study used facility-based, cross-sectional design and quantitative approach to assess readiness for the management of HTN and diabetes. All the 16 HFs at the Health Centre III (HCIII) level in Bidibidi were studied, and a sample size of 148 healthcare workers (HCWs) was determined using Yamane's formula (1967). Proportionate sample sizes were determined at each HF and the simple random sampling technique was used. HF data were collected using the Service Availability and Readiness Assessment (SARA) checklist and a structured questionnaire used among HCWs. Data were analyzed using SPSS version 20. Univariate analysis involved descriptive statistics; bivariate analysis used chi-square, Fisher's exact test, and multivariable regression analysis for readiness of HCWs.

**Results:**

16 HCIIIs were studied in five zones and involved 148 HCWs with a mean age of 28 (std ±4) years. The majority 71.6% (106) were aged 20–29 years, 52.7% were females, and 37.8% (56/148) were nurses. Among the 16 HFs, readiness average score was 71.7%. The highest readiness score was 89.5% while the lowest was 52.6%. The 16 HFs had 100% diagnostic equipment, 96% had diagnostics, and 58.8% had essential drugs (low for nifedipine, 37.5%, and metformin, 31.2%). Availability of guidelines for the management of HTN and diabetes was 94%, but only low scores were observed for job aid (12.5%), trained staff (50%), and supervision visits (19%). Only 6.25% of the HFs had all the clinical readiness parameters. On the other hand, only 24% (36) of the HCWs were found to be ready to manage HTN and diabetes cases. Chi-square tests on sex (*p* < 0.001), education level (*p*=0.002), and Fisher's tests on profession (*p* < 0.001) established that HCWs with bachelor's degree (AOR = 3.15, 95% CI: 0.569–17.480) and diploma (AOR = 2.93, 95% CI: 1.22–7.032) were more likely to be ready compared to the reference group (certificate holders). Medical officers (AOR = 4.85, 95% CI: 0.108–217.142) and clinical officers (AOR = 3.79, 95 CI: 0.673–21.336) were more likely to be ready compared to the reference group, and midwives (AOR = 0.12, 95% CI: 0.013–1.097) were less likely to be ready compared to the reference group. In addition, female HCWs were significantly less likely to be ready compared to male HCWs (AOR = 0.19, 95% CI: 0.073–474).

**Conclusion:**

HFs readiness was high, but readiness among HCWs was low. HFs had high scores in equipment, diagnostics, and guidelines, but essential drugs, trained staff, and supervision visits as well HCWs had low scores in trainings and supervisions received. Being male, bachelor's degree holders, diploma holders, medical officers, and clinical officers increased the readiness of the HCWs.

## 1. Background

HTN and diabetes are the leading lifestyle diseases and common causes of the rapid increase in NCDs [1]. NCDs are the greatest global contributors to mortality, morbidity, and a major health challenge of the 21st century. The global burden of NCDs remains unacceptably high; in 2016, NCDs were responsible for 41 million of the world's 57 million deaths (71%). 15 million of these were premature deaths between 30 and 70 years [2–5], with the burden greatest in low- and middle-income countries (LMICs) where 78% of all NCD deaths and 85% of premature deaths occurred [3].

NCDs account for nearly 86% of deaths and 77% of the disease burden in Europe [4]. African (22%), Eastern Mediterranean (24%), and Southeast Asian (23%) regions have high probability of premature adult NCD mortality compared to American (15%), European (17%), and Western Pacific (16%) regions [3]. The probability of dying from any of the NCDs between exact age 30 and 70 in Africa was the highest in Sierra Leone (30.5%), followed by 13.4% in Kenya and 21.9% in Uganda according to World Health Statistics 2018 Report on monitoring SDG 3, target 3.4 [2].

In Uganda, emerging evidence from empirical studies estimates that NCDs account for 11–13% of the disease burden [6] and in 2016, NCDs were estimated to account for 33% of all deaths with the risk of premature death due to NCDs, the highest in Uganda [3, 7]. Uganda has high prevalence of HTN (24%) and diabetes (3.4%) [6, 8] and both conditions are among the top 20 causes of outpatient department attendance, admission, and mortality [9]. There is also a high prevalence of risk factors; overweight (14.5%), obesity (4.6%), physical inactivity (4.3%), current tobacco users (11%), and alcohol consumption (28.9%) [6, 8]. It was also estimated that 3.2% of Ugandans have diabetes and 6% have CVDs [7]. Given the above statistics, refugees in their settlements in Uganda may also be affected, with the problems of HTN and diabetes as crises interrupt the normal coordinated systems of care for chronic diseases, which are needed to prevent, detect, monitor, treat, and manage the diseases and their implications.

In Uganda, 55% of the refugee households, including those with chronic illnesses, lacked access to health services; 56% of these are in West-Nile region. In Yumbe-Bidibidi, 61% of the refugee households were in need of health services including for NCDs [10]. Data on NCDs in Bidibidi are scarce; however, HFs row data indicate CVDs (54.3%) and metabolic disorders (20.6%) were the leading causes of consultations among the major NCDs [11]; unpublished).

HFs readiness is an important indicator for NCD service delivery, and WHO [12] developed SARA as an annual monitoring system for service delivery, but no such an assessment has been done in the Bidibidi settlement. Without appropriately addressing the current NCD challenges, NCDs may lead to more morbidity, mortality, and economic burden associated with their management or complications among refugees. Therefore, this study determined the readiness of primary health facilities in managing HTN and diabetes in the Bidibidi settlement, which may inform strategies for efficient and effective management of NCDs, thus increasing access to hypertensive and diabetic care.

## 2. Methods

This study was conducted in the Bidibidi refugee settlement in Yumbe district, which is situated in the Northwestern (West Nile) region of Uganda bordered by Maracha and Koboko districts in the west; Arua district in the south; Moyo district in the east, and South Sudan to the north. The district consists of 3 counties (Aringa Central, Aringa North, and Aringa South), 3 health subdistricts, 13 subcounties, 101 parishes, 636 villages, and 46 functional HFs (1 hospital, 2 HCIVs, 26 HCIIIs, and 17 HCIIs); the ownership was as follows: 2 7 government, 3 private not-for-profit (PNFP), and 16 nongovernmental organizations (NGOs) -UNHCR. The study setting, the Bidibidi refugee settlement, is the largest settlement in Uganda. It was opened in July 2016 following a fresh outburst of civil war/crises in South Sudan, and hosts 223,939 refugees [13]. Its administrative units are divided into 5 zones and 75 villages, and there are 16 HCIIIs providing primary health care (PHC) services in the settlement.

We utilized facility-based cross-sectional study design and quantitative approach to assess readiness for the management of HTN and diabetic patients at all the 16 HFs in the Bidibidi settlement. A sample size of 148 healthcare workers (HCWs) from all the 16 HFs was determined using Yamane's formula [14], with 5% (0.05) acceptable margin of error. Proportionate sample sizes were determined at each facility and simple random sampling technique was used.

An adapted WHO [12] SARA tool and a self-administered questionnaire were used among HFs and HCWs, respectively, to collect data; in addition, the HF readiness domain tracer items were observed. The HF SARA tool collected data on the availability of diagnostic equipment, essential medicine, diagnostics, guidelines, job aid, trained HCWs, and supervision status. The self-administered questionnaire for HCWs collected information on their knowledge, experience, confidence, in-service training, and supervision received to manage HTN and diabetes cases. Preceding and during data collection was training and supervision of the research assistants, respectively, to ensure the quality of data. HF questionnaires were administered at HFs by the trained research assistants and questionnaires for HCWs were self-administered. Those of the HCWs who were absent were followed to complete the questionnaire.

All the completed questionnaires were reviewed on a daily basis for accuracy, completeness, and consistency, and any errors or inconsistencies highlighted were fixed prior to analysis. The collected data were double entered in Microsoft Excel and exported to Statistical Package for Social Science (SPSS) version 20 for univariate, bivariate, and multivariable regression analysis at 95% confidence intervals and adjusted odds ratios were recorded. Frequencies and percentages were calculated for categorical variables and measures of central tendency for continuous variables. HFs and HCWs were kept anonymous to ensure confidentiality; instead, serial numbers were used during data analysis and presentation. The analyzed data were presented in tables and figures.

The dependent outcome, the HFs readiness, was determined by the mean scores of the technological readiness domains with 13 items and clinical readiness domains with 6 items using the WHO (2015) SARA approach, and a cut-off point at ≥70% was adopted as the readiness score from a study in Zambia [15] as WHO SARA had no readiness cut-off point. On the other hand, readiness of HCWs was based on knowledge (2 items), experience (2 items), confidence (2 items), and in-service training (2 items) for both HTN and diabetes, and supervision (1 item) received on NCDs (HTN and diabetes), where ≥7 out of 9 was considered as the readiness score to manage HTN and diabetes cases.

The independent variables for HFs readiness were the basic equipment with 5 items, essential drugs with 5 items, diagnostics with 3 items, guidelines with 2 items, job aid with 2 items, trained staff with 01 item, and supervision status with 01 item; on the other hand, the independent variables for readiness of HCWs were knowledge measured using 10 components of questions (those who scored ≥8 correct answers were considered to have good knowledge, 5–7 fair and ≤4 poor), experience, confidence, in-service training, and supervision as described in Table 1.

A descriptive analysis was undertaken for numerical and categorical data whose frequencies and subsequent percentages were also computed. Furthermore, figures and tables were processed using MS Excel. A chi-squared test for categorical and nominal variables was carried out to establish the associations between readness of HFs and HCWs to manage HTN and diabetes. On the other hand, for cell values less than 5, Fisher's exact test was performed, and the subsequent values and probability values were recorded. The level of knowledge, experience, confidence of HCWs, their in-service training, and supervision status were each cross-tabulated with sociodemographic variables. In the multivariate regression analysis, adjustments were done on three variables (sex, education level, and profession) of HCWs that had a significant association with the readiness to control confounders and determine the main variables (socio-demographic variables) responsible for readiness of the HCWs where adjusted odds ratios and 95% confidence interval were reported.

This study was approved by the Clarke International University Research Ethics Committee. Permission was obtained from the Office of the Prime Minister (OPM), District Health Office (DHO), International Rescue Committee (IRC), Médecins Du Monde (MDM), and Save the Children International (SCI) - Yumbe district. Informed consent was obtained from the HF in-charges and HCWs participating in the study after being informed about their rights, benefits/risks, and obligations to withdraw from the study, and all respondents gave a written informed consent before participation.

## 3. Results

### 3.1. Health Facilities' Readiness to Manage HTN and Diabetes Cases

#### 3.1.1. Background Characteristics of the Bidibidi Settlement and Health Facilities

In Table 2, the Bidibidi refugee settlement has 5 zones and 16 HCIIIs from which data for this study were collected. Most (15) of the HFs were private and 01 government/private in a rural setting.

#### 3.1.2. Readiness of the Health Facilities in Managing HTN and Diabetes Cases

**Figure 1:**
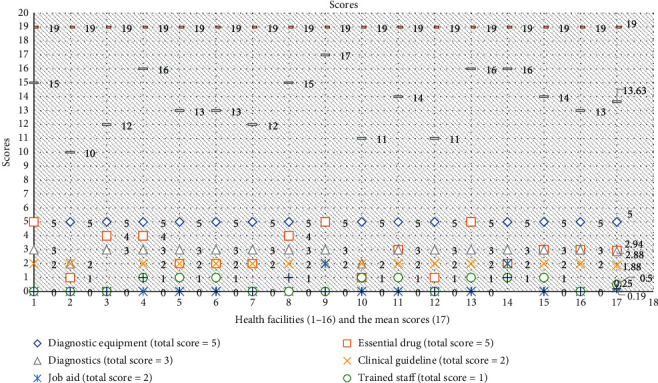
HFs' readiness to manage HTN and diabetes (parameters, total maximal score, and total actual scores).

In Table 3 and Figure 1, the 16 HFs' readiness to manage HTN and diabetes calculated from the mean scores of the technological and clinical readiness domains was 71.7%; HFs with a higher level of readiness above the mean score were 50%, with the highest facility scoring 89.5% and the lowest scoring 52.6%. Technologically, all the HFs had 100% functional diagnostic equipment (sphygmomanometer, stethoscope, weighing scale, glucometer, and glucometer stripes) assessed; availability of diagnostics (blood sugar test, urine dipstick-protein test, and urine dipstick-ketones test) conducted on-site was 96%, availability of essential drugs (bendroflumethiazide, nifedipine, methyldopa, metformin, and glibenclamide) was low at 58.8%, 18.8% of the HFs had all the 5 essential drugs assessed, and 43.8% had 1-2 of the assessed essential drugs available at the time of assessment. Clinically, availability of guidelines for the diagnosis and treatment of HTN and diabetes was 94%; however, availability of job aid (12.5%), trained staff (50%), and supervision visits (19%) had low scores. Only 6.25% of the HFs had all the clinical readiness parameters assessed. Job aid, training, and supervision were consistently lacking across facilities. The highly ready HFs were government/private owned, probably due to consolidated efforts in support of the HF. Figure 1 was used to show the parameter, total maximal scores, and total actual scores.

### 3.2. Readiness of Healthcare Workers to Manage HTN and Diabetes Cases

In Table 4, the mean age of HCWs was 28 years (Std ± 4 years), the minimum age was 22 years, the maximum was 45 years, and the majority, 71.6% (106), were aged 20–29 years, meaning most of them were still young in their 20s. The study shows that the number of HCWs decreased with increase in years probably because older HCWs prefer stability and job security, and mostly join government employment schemes compared to nongovernmental organization (NGO) jobs. More than half, 52.7% (78), of the HCWs were females; majority, 67.6% (100), of the HCWs were married; more than half, 60.1% (89), of the HCWs had attained certificate level of education, and by cadre, 37.8% (56) of the HCWs were nurses, followed by midwives, 34.5% (51) (see Table 4).

#### 3.2.1. Readiness of Healthcare Workers to Manage HTN and Diabetes Cases

In Table 5, knowledge of the HCWs on HTN and diabetes was measured using ten questions. The HCWs who scored ≥8 correct answers were considered to have good knowledge, 5–7 as fair, and ≤4 as poor; 85.8% (127) and 62.2% (92) of HCWs had good knowledge on HTN and diabetes, respectively. The result revealed that more than half of the HCWs reportedly had experience in the management of HTN and diabetes, accounting for 65.5% (97) and 53.4% (79), respectively. Regarding confidence in the management of HTN and diabetes, the majority of the HCWs reported better level of confidence for the management of HTN than diabetes management, accounting for 81.1% and 68.2%, respectively. In terms of in-service training of HCWs on HTN and diabetes management, the finding revealed that less than a quarter, 23.6% and 23.4%, of the HCWs were trained on HTN and diabetes management, respectively, in the last two years. Supervision of health workers by their supervisors is important to ensure adherence to the established standard operating procedures as well as the use of guidelines. However, this study found that less than a quarter (15.5%) of the HCWs reported to have been supervised on NCD (HTN and diabetes) management in the last three months.

#### 3.2.2. Level of Readiness to Manage Hypertension and Diabetes Cases among Healthcare Workers

**Figure 2:**
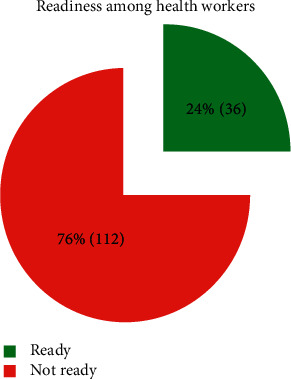
Level of readiness among healthcare workers.

Figure 2 shows that the individual level of readiness of the 148 HCWs was measured as those who scored ≥7 of the 9 parameters used to measure readiness as good knowledge, had experience, self-reported confidence, and received in-service training on HTN and diabetes management and supervision status on NCDs including HTN and diabetes. The level of readiness among HCWs was low at 24% (36), whereas 76% (112) of the HCWs were not ready based on the scores of the above parameters. Figure 2 also shows an aggregate analysis of Table 5 where the individual level of readiness of the 148 HCWs was measured; those who scored ≥7 of the 9 parameters used to measure readiness as good knowledge, had experience, self-reported confidence, and received in-service training on HTN and diabetes management and supervision status on NCDs including HTN and diabetes. The level of readiness among HCWs was 24% (36), which is low, whereas 76% (112) of the HCWs were not ready based on the scores of the above parameters, especially on in-service training, supervision, level of experience, and confidence in the management of diabetes cases.

In Table 6, sex, education level, and profession of the HCWs had a statistically significant relationship with the level of readiness, whereas age had no statistically significant relationship with the readiness of HCWs. The study found that 83 (78.3%) out of 106 HCWs aged 20–29 years were ready to manage both HTN and diabetes cases. This study found no statistically significant relationship between readiness of HCWs and their age category (Fisher's = 3.441, *p*=0.137). Regarding sex of the HCWs, male HCWs were found to be more ready than females accounting for 42 (60.0%) males out of 70 and 7 (9.0%) females out of 78. There was a statistically significant relationship between readiness of HCWs and their sex (*χ*^2^ = 19.67, d*f* = 1, *p* < 0.001). Concerning the education level, this study found that HCWs with certificate level of education were more than any other level. However, out of 35 HCWs who were ready, 20 of them were those who attained diploma level of education. It also emerged that there was a statistically significant relationship between the level of education and the readiness of the HCWs (*χ*^2^ = 12.84, d*f* = 2, *p*=0.002). Looking at profession of the HCWs, clinical officers (19 out of 37) and nurses (13 in 56) were found to be ready to manage HTN and diabetes. Similarly, there was a statistically significant relationship between the profession and the readiness of HCWs (Fisher's = 33.27, *p* < 0.001).

Table 7 indicates that female HCWs were less likely to be ready to manage HTN and diabetic cases (AOR = 0.19, 95% CI: 0.073–474). The finding further implies that being a female HCW increases readiness by19%. Regarding education, HCWs with bachelor's degree level of education were more likely to be ready to manage HTN and diabetic cases (AOR = 3.15, 95% CI: 0.569–17.480). The finding further implies that being a bachelor's degree holder increases an HCW's readiness by 315%, whereas diploma holders were 2.93 times more likely to be ready compared to the reference group (AOR = 2.93, 95% CI: 1.22–7.032). Concerning the profession of HCWs, medical officers were found to be 4.85 times more likely to be ready compared to other professions (AOR = 4.85, 95% CI: 0.108–217.142). Clinical officers were 3.79 times more likely to be ready compared to the reference group (AOR = 3.79, 95 CI: 0.673–21.336). Similarly, midwives were 0.12 times less likely to be ready compared to the reference group (AOR = 0.12, 95% CI: 0.013–1.097).

## 4. Discussion

### 4.1. HFs' Readiness to Manage HTN and Diabetes

This study determined the readiness of HFs in managing HTN and diabetes in primary health facilities in the Bidibidi settlement. HFs' readiness is an important indicator for NCD service delivery. We found that the Bidibidi settlement HFs' readiness was high and the highly ready HF was NGO/government owned. Only limited service readiness evaluation studies have been conducted in a refugee setting in Africa with which this study has to be compared; however, some of the findings were compared with proxy studies in nonrefugee settings. Although WHO [12] SARA did not set a cut-off point for HFs readiness, this study found the HFs' readiness was slightly above the readiness mean score cut-off point of 70% considered for HFs in Zambia in a nonrefugee setting [15].

The 2030 sustainable development goals (SDGs) agenda recommend targets to prevent and control NCDs such as CVDs and diabetes; however, weak health systems may affect HFs' readiness in many countries [2]. In crisis settings, the level of disruption to service delivery impedes these efforts.

This study also documented the level of the HFs' technological readiness domains that contributed to the good level of HFs' readiness in the settlement. These included the availability of diagnostic equipment, which was above the WHO recommended 80% availability level for affordable basic technologies required to treat major NCDs in HFs [16], implying well-equipped HFs to diagnose and treat HTN and diabetes in the settlement; availability of diagnostics was also high, implying the capacity of the HFs to carry on-site basic diagnostic tests such as blood sugar and urine tests.

Katende et al. [17] similarly found HCIIIs with very high (92%) basic diagnostic equipment in Uganda; however, Peck et al. [18] found that only 70.8% of HFs had diagnostic equipment in Tanzania. On the other hand, Katende et al. [17] found that hospitals had a better level of availability of diagnostic equipment compared to the lower facilities. However, this study focused on HCIIIs in the refugee settlement. Although this study found that all the HFs had diagnostic equipment available for HTN and diabetes, earlier findings by Peck et al. [18] suggest that less than half of the studied HFs with diagnostic equipment accounted for only 33.3% of the diabetes patients. Our study did not list the height measurement device in the list of the equipment, but Peck et al. [18] registered 70% (14/20) availability of the device.

In Uganda, antihypertensives of bendroflumethiazide, nifedipine, and methyldopa are the first-line medicines at HCIIIs depending on the type and stage of HTN, while metformin and glibenclamide are the respective first- and second-line medicines for diabetic care. According to MoH guidelines, metformin must be available for patients at HCIIIs [6]. However, we found that the availability of essential drugs was generally low, especially nifedipine and metformin, that is, lower than 80% of the WHO availability target for NCD management in HFs [16]. This implies both low readiness and poor access to HTN and diabetes care services at HFs, as some patients may leave the HFs without essential drugs. This study's finding on essential drug availability was slightly conforming to that of Adinan et al. [19] who found that the overall availability of essential medicines in HFs was as low as 41.2% and was 50% at HCs in Tanzania. This contributes negatively toward scores of HFs' readiness to manage HTN and diabetes and indicates a gap in the pharmaceutical supply chain.

In terms of clinical readiness domains, a high number of HFs had guidelines for diagnosis and treatment of HTN and diabetes. The primary tool was the Uganda clinical guideline, [20] which was distributed to the HFs to improve the clinical practices of clinicians. The level of availability of guidelines for managing HTN and diabetes in this study was found to be higher in comparison to other similar studies carried out elsewhere. For instance, Adinan et al. (2019) [19] established 76% availability in Tanzania, and Katende et al. (2015) established 75% readiness at Health Centre IIIs in Uganda. However, the level of readiness noted above greatly differs from findings in other studies conducted by Peck et al. (2014) [18] who observed only 13% in HFs in Tanzania and MoHP Nepal (2018) [21] that observed 1.4% and 4% for both CVDs and diabetes, respectively, in Nepal.

The study also noted that Bidibidi HFs lacked job aid for information, education, and communication displayed at the HFs for patients and mass population education on HTN and diabetes (risk communication). This implies that the Bidibidi community may lack knowledge and awareness on basic facts on HTN and diabetes. This finding conforms to the reports from Nigeria by Okpetu et al. [22] who noted insufficient job aid in PHC facilities on NCDs.

Supervision at the HF level for monitoring is important for the identification of gaps and challenges being faced by HFs and HCWs, so that mentoring and trainings can be planned. We found exceptionally low scores on supervision visits at HFs with at least a trained staff for the management of HTN and diabetes. Lack of training, mentoring, monitoring, and support supervision has a direct impact on the quality of services provided by the health staff who may lack the required knowledge and competences for NCD care. In this study, fewer HFs had trained staff compared to the finding in a nonrefugee study setting by Adinan et al. [19] in Tanzania where 91.7% of health centers had trained staff, more than Biswas et al. [23] who reported 18.8% and 14.7% of the HFs had trained staff for providing diabetes and CVD services, respectively. However, a study in Uganda by Katende et al. [17] found that none of the HCIIIs had training for their HCWs.

The findings with regard to HFs that received supervision in the last three months were close to the results of Peck et al. [18] in Tanzania where 20.8% (5/24) of the HFs reported receiving a monitoring and supervision visit in the past 3 months. Katende et al. [17] also reported support supervision visits to HCIIIs at only 8.3% of HFs in Uganda.

### 4.2. HCWs' Readiness to Manage HTN and Diabetes

HCWs provide soft skills in the management of HTN and diabetes at HFs, and their readiness is important in determining the outcome of the care. In this study, we found the mean age of HCWs was 28 years (Std ± 4 years) and few HCWs were older than 30 years. We found that most of the HCWs were not ready given the lack of knowledge, experience, confidence, in-service training, and supervision received on case management. However, we did not find HCWs' readiness evaluation studies conducted in a refugee setting in Africa to make meaningful comparisons.

We found that most of the HCWs had good knowledge, more experience, and more confidence on HTN management compared to diabetes. This may be due to the differences in understanding the pathophysiology and management of the two conditions where HTN was well understood or prioritized by most HCWs. This may further impact negatively on the level of attention and care toward diabetic cases. However, we had also acknowledged and taken account of interpreting results of self-reported information on HCWs' readiness parameters as a limitation of the study.

In this study, HCWs who scored ≥8 correct answers out of 10 were considered to have good knowledge in the management of HTN and diabetes. Results from this study revealed that more than half of the study participants had experience in management of HTN and diabetes accounting for 65.5% (97) and 53.4% (79), respectively. Overall, these findings are similar to those in a study conducted by Peck et al (2014) [18] in Tanzania where HWCs' knowledge on HTN was 59% (198) and 56% (187) for diabetes, respectively. While Adinan et al (2019) [19] carried out another study in Tanzania which established HTN ((69.4%) 43) and diabetes ((41.7%) 25) readiness. Similarly, Biswas et al (2018) [23] reported much lower percentage on HCWs' readiness in Bangladesh. According to the findings, HTN was 14.7% while diabetes was 18.8%. HCWs who received supervision on NCDs were few, but no study reported on the percentage of HCWs supervised at the HFs. The differences in findings from this study and those in studies conducted in Tanzania and Bangladesh could be attributed to differences in study settings and levels of the HCs as well as level of professional training of the HCWs.

We also found that sex, level of education, and profession of the HCWs were statistically significant to readiness, and being male, bachelor's degree holders, diploma holders, medical officers, and clinical officers increases the readiness of the HCWs.

### 4.3. Study Limitation

This study assessed HCIIIs in the Bidibidi refugee settlement using the quantitative approach. Hence, it had limitations compared to other HF levels and qualitative study aspects, and we recommended a further mixed method research with the inclusion of other HF tiers and ownership to assess the quality of services for the management of HTN and diabetes cases. We had also acknowledged and taken account of interpreting results of self-reported information on HCWs' readiness parameters as a limitation to this study.

## 5. Conclusion

Bidibidi HFs' readiness to manage HTN and diabetes cases was high and readiness among HCWs was low; this calls for capacity-building interventions, such as training, mentoring, monitoring and supervision, to increase the HCWs' readiness. These study findings are imperative in programming and scaling-up of NCD policy and interventions in humanitarian settings, especially in sub-Saharan Africa.

## 6. Recommendations

The HF management should conduct similar analyses periodically (biannually or annually) in order to identify gaps and improve service quality.

The HF management should ensure that diagnostic equipment are monitored and continue to be maintained in the right quantity and quality.

The HF management should ensure that essential drugs for HTN and diabetes management are made available at the HFs in the right quantity and type.

The health authority/administration should conduct periodic refresher trainings and supervision of HCWs on HTN and diabetes management.

## Figures and Tables

**Table 1:** Background characteristics of health facilities in the study site (study variables and their measurements).

*Parameter*	*Parameter description*
Readiness	State of being prepared to manage HTN and diabetes cases.

*HFs, technological readiness domains*	
Basic equipment	Observed functional sphygmomanometer, stethoscope, weighing scale, glucometer, and glucometer stripes available in the service area or clinic.
Essential medicine	Observed valid medicine listed for use at HCIII level to manage HTN and diabetes available in the service area or clinic (HTN: Bendroflumethiazide, nifedipine, and methyldopa; Diabetes: metformin and glibenclamide).
Diagnostic	Observed the ability to conduct on-site tests for blood sugar, urine (protein and ketones) in the service area or clinic.

*HFs, clinical readiness domains*	
Guideline	Observed diagnosis and treatment guidelines for HTN and diabetes available in the service area or clinic.
Job aid	Observed Information, Education and Communication (IEC) materials on HTN and diabetes displayed in the clinic.
Trained staff	Facility has at least one HCW trained on HTN and diabetes management in the last 2 years.
Supervision	Facility received at least one supervision visit from the higher level (health management team) in the last 3 months.

*HCWs, readiness domains*	
Knowledge	HCW scored ≥8/10 when assessed using case scenarios/questions on HTN and diabetes management.
Experience	HCW self-reported managing at least ≥5 cases each of HTN and diabetes in the last three months.
Confidence	HCW self-reported being confident in managing HTN and diabetes.
Training	HCW self-reported receiving an in-service training on HTN and diabetes management in the last two years.
Supervision	HCW self-reported receiving supervision on NCDs' (HTN and diabetes) management in the last three months.

**Table 2:** Readiness of the health facilities.

Bidibidi settlement zones	Number of HFs per zone	Levels of the HFs	HF ownership	HF setting
Zone I	03	All HCIIIs	Private	Rural
Zone II	03	All HCIIIs	Private	Rural
Zone III	04	All HCIIIs	03 private, 01 government/private	Rural
Zone IV	03	All HCIIIs	Private	Rural
Zone V	03	All HCIIIs	Private	Rural

**Table 3:** Readiness of the health facilities.

Health facilities (HCIII levels)	Technological readiness domains			Clinical readiness domains				Readiness	
	Diagnostic equipment (total score = 5)	Essential drug (total score = 5)	Diagnostics (total score = 3)	Clinical guideline (total scores = 2)	Job aid (total score = 2)	Trained staff (total score = 1)	Supervision (total score = 1)	Total maximal scores = 19	Total actual scores
1	5	5	3	2	0	0	0	19	15
2	5	1	2	2	0	0	0	19	10
3	5	4	3	0	0	0	0	19	12
4	5	4	3	2	0	1	1	19	16
5	5	2	3	2	0	1	0	19	13
6	5	2	3	2	0	1	0	19	13
7	5	2	3	2	0	0	0	19	12
8	5	4	3	2	0	0	1	19	15
9	5	5	3	2	2	0	0	19	17
10	5	1	2	2	0	1	0	19	11
11	5	3	3	2	0	1	0	19	14
12	5	1	3	2	0	0	0	19	11
13	5	5	3	2	0	1	0	19	16
14	5	2	3	2	2	1	1	19	16
15	5	3	3	2	0	1	0	19	14
16	5	3	3	2	0	0	0	19	13
Mean scores	5.00 (100%)	2.94 (58.8%)	2.88 (96.00%)	1.88 (94.00%)	0.25 (12.50%)	0.50 (50.00%)	0.19 (19.00%)	19 (100%)	13.63 (71.7%)

**Table 4:** Sociodemographic characteristics of healthcare workers.

Variable	Frequency (*n* = 148)	Percentage (100%)
Age category (years)		
20–29	106	71.6
30–39	36	24.3
40–49	6	3.1

Sex		
Male	70	47.3
Female	78	52.7

Marital status		
Single	47	31.8
Married	100	67.6
Divorced/separated	1	0.7

Education level		
Certificate	89	60.1
Diploma	52	35.1
Bachelor's	7	4.7
Master's		

Profession/cadre		
Nurse	56	37.8
Midwife	51	34.5
Clinical officer	37	25.0
Medical officer	4	2.7

**Table 5:** Univariate analysis on healthcare workers' readiness parameters.

Variables	Responses	Frequency (*n*)	Percentage
Knowledge to manage cases of			
HTN	Good	127	85.8
	Fair	20	13.5
	Poor	1	0.7
Diabetes	Good	92	62.2
	Fair	54	36.5
	Poor	2	1.4

Experience of having managed ≥5 cases in the last 3 months			
HTN	Yes	97	65.5
	No	51	34.5
Diabetes	Yes	79	53.4
	No	69	46.6

Confidence in managing cases of			
HTN	Yes	120	81.1
	No	28	18.9
Diabetes	Yes	101	68.2
	No	47	31.8

Training (in-service) received in the last two years			
HTN	Yes	35	23.6
	No	113	76.4
Diabetes	Yes	33	22.3
	No	115	77.7

Supervision of NCD services in the last three months			
Received supervision on NCD services	Yes	23	15.5
	No	125	84.5

**Table 6:** Bivariate analysis between readiness among healthcare workers and sociodemographic characteristics.

Variable	Readiness of healthcare workers		Total	Fisher's/chi-square (*χ*^2^), degree of freedom (d*f*)	*p* value
	Not ready	Ready
Age category (years)				3.441	0.137
20–29	23 (21.7%)	83 (78.3%)	106		
30–39	12 (33.3%)	24 (66.7%)	36		
40–49	0 (0.0%)	6 (100%)	6		
Sex				19.67 (1)	<0.001^*∗∗*^
Male	28 (40.0%)	42 (60%)	70		
Female	71 (91.0%)	7 (9.0%)	78		
Education level				12.84 (2)	0.002^*∗*^
Certificate	77 (86.5%)	12 (13.5%)	89		
Diploma	32 (61.5%)	20 (38.5%)	52		
Bachelors	4 (57.1%)	3 (42.9%)	7		
Profession/cadre				33.27	<0.001^*∗∗*^
Nurse	43 (76.8%)	13 (23.2%)	56		
Midwife	50 (98.0%)	1 (2.0%)	51		
Clinical officer	18 (48.6%)	19 (51.4%)	37		
Medical officer	2 (50.0%)	2 (50.0%)	4		

*p* < 0.001^*∗∗*^; *p* < 0.005^*∗*^.

**Table 7:** Multivariable analysis for the association between sociodemographic factors and readiness.

Variable	Readiness of healthcare workers		Crude odds ratio, 95% CI	Adjusted odds ratio, 95% CI
	Not ready (%)	Ready (%)
Sex				
Male	28 (40.0)	42 (60)	1	1
Female	71 (91.0)	7 (9.0)	0.15 (0.059–0.368)	0.19 (0.073–474)
Education level				
Certificate	77 (86.5)	12 (13.5)	1	1
Diploma	32 (61.5)	20 (38.5)	4.01 (1.756–9.160)	2.93 (01.22–7.032)
Bachelor's	4 (57.1)	3 (42.9)	4.81 (0.956–24.216)	3.15 (0.569–17.480)
Profession				
Nurse	43 (76.8)	13 (23.2)	1	1
Midwife	50 (98.0)	1 (2.0)	0.07 (0.008–0.527)	0.12 (0.013–1.097)
Clinical officer	18 (48.6)	19 (51.4)	3.49 (1.427–8.542)	3.79 (0.673–21.336)
Medical officer	2 (50.0)	2 (50.0)	3.31 (0.423–25.843)	4.85 (0.108–217.142)

*p* < 0.001^*∗∗*^; *p* < 0.005^*∗*^.

## Data Availability

The data sets for this study are available upon request to the corresponding author.

## Abbreviations

CVDs: Cardio vascular diseases
DHO: District health office
HC: Health center
HCW(s): Healthcare worker(s)
HF(s): Health facility(ies)
HTN: Hypertension
IRC: International Rescue Committee
LMICs: Low- and middle-income countries
MDM: Médecins Du Monde
MOH: Ministry of Health
NCD(s): Noncommunicable disease(s)
NGO: Nongovernmental organization
OPM: Office of the Prime Minister
PHC: Primary healthcare
SARA: Service availability and readiness assessments
SCI: Save the Children International
SDG: Sustainable development goals
STD: Standard deviation
UNHCR: United Nations High Commission for Refugees
WHO: The World Health Organization.
